# Synthetic RGDS peptide attenuates lipopolysaccharide-induced pulmonary inflammation by inhibiting integrin signaled MAP kinase pathways

**DOI:** 10.1186/1465-9921-10-18

**Published:** 2009-03-09

**Authors:** Changsuk Moon, Jeong Ran Han, Hyun-Jung Park, Jong Sik Hah, Jihee Lee Kang

**Affiliations:** 1 Department of Physiology, Division of Cell Biology, Ewha Medical Research Center, School of Medicine, Ewha Womans University, Seoul, Seoul, Korea

## Abstract

**Background:**

Synthetic peptides containing the RGD sequence inhibit integrin-related functions in different cell systems. Here, we investigated the effects of synthetic Arg-Gly-Asp-Ser (RGDS) peptide on key inflammatory responses to intratracheal (*i.t.*) lipopolysaccharide (LPS) treatment and on the integrin signaled mitogen-activated protein (MAP) kinase pathway during the development of acute lung injury.

**Methods:**

Saline or LPS (1.5 mg/kg) was administered *i.t. *with or without a single dose of RGDS (1, 2.5, or 5 mg/kg, i.p.), anti-α_v _or anti-β_3 _mAb (5 mg/kg, i.p.). Mice were sacrificed 4 or 24 h post-LPS.

**Results:**

A pretreatment with RGDS inhibited LPS-induced increases in neutrophil and macrophage numbers, total protein levels and TNF-α and MIP-2 levels, and matrix metalloproteinase-9 activity in bronchoalveolar lavage (BAL) fluid at 4 or 24 h post-LPS treatment. RGDS inhibited LPS-induced phosphorylation of focal adhesion kinase and MAP kinases, including ERK, JNK, and p38 MAP kinase, in lung tissue. Importantly, the inhibition of the inflammatory responses and the kinase pathways were still evident when this peptide was administered 2 h after LPS treatment. Similarly, a blocking antibody against integrin α_v _significantly inhibited LPS-induced inflammatory cell migration into the lung, protein accumulation and proinflammatory mediator production in BAL fluid, at 4 or 24 h post-LPS. Anti-β_3 _also inhibited all LPS-induced inflammatory responses, except the accumulation of BAL protein at 24 h post-LPS.

**Conclusion:**

These results suggest that RGDS with high specificity for α_v_integrins attenuates inflammatory cascade during LPS-induced development of acute lung injury.

## Background

Acute lung injury is characterized by an intense pulmonary inflammatory response, with neutrophil recruitment, interstitial edema, disruption of epithelial integrity, and parenchymal injury [1]. The migration of leukocytes to inflamed sites depends on the interactions of various integrins expressed on leukocytes with the Ig superfamily of cell adhesion molecules (ICAM-1 and ICAM-2) present on the endothelium and with the extracellular matrix ligands. Multiple integrins participate in neutrophil migration into the lung during LPS-induced lung injury. In addition to β_2 _integrin (CD18), α_4_β_1 _and α_5_β_1 _integrins also contribute to β_2_-independent neutrophil migration during pulmonary inflammation [2]. However, neither β_2 _blockade nor α_4 _plus α_5 _blockade reduced neutrophil accumulation in the parenchyma and only minimally in the airspaces [2]. These findings suggest that there are functionally redundant alternative integrin pathways that signal neutrophil recruitment to the lung and inflammatory responses.

Many members of the integrin family, such as α_5_β_1_, αIIβ_3_, α_v_β_3_, α_v_β_6_, α_8_β_1_, α_v_β_1_, α_v_β_5_, and α_v_β_8_, recognize the Arg-Gly-Asp (RGD) motif within their ligands [3], which include fibronectin, fibrinogen, vitronectin, von Willebrand factor and collagens [4]. Synthetic peptides containing the RGD sequence have been shown to compete with adhesive proteins for binding to these integrin receptors [5], and thus, inhibit integrin-related functions in different cell systems. This suggests the possibility that therapeutic RGD-containing integrin ligands could be developed to ameliorate inflammatory cascades, specifically those that cause the migration and activation of leukocytes.

Several previous reports suggest the possibility that RGDS alters systemic inflammation. RGDS (i.p.) effectively inhibits collagen-triggered activation of leukocytes and platelets, such as aggregation and oxygen radical production [6]. Koning et al. [7] demonstrated binding of RGD peptide to proliferating vascular endothelial cells in LPS-induced inflammation in rats. Moreover, RGD peptide inhibits the expression of inflammatory cytokines, iNOS, and MMP-9 in rat liver after cold ischemia/reperfusion injury [8]. However, the local effect of RGDS on pulmonary inflammation has not been evaluated.

The inhibitory effect of RGD peptide on LPS-induced mitogen-activated protein (MAP) kinase activity and TNF-α production was shown in RAW 264.7 cell model, suggesting that LPS-induced integrin signaling converges on MAP kinases [9]. Here, we evaluated the effects of RGDS peptide on the pulmonary inflammatory responses, including leukocyte migration into the lungs, damage to the lung blood-gas barrier, and proinflammatory mediator production following *i.t. *LPS treatment. Furthermore, we also examined the effects of RGDS on the integrin signaled MAP kinase pathway during the development of acute lung injury.

## Materials and methods

### Reagents

LPS (*Escherichia coli *lipopolysaccharide, 055:B5), Arg-Gly-Asp-Ser (RGDS) and Arg-Gly-Glu-Ser (RGES) were purchased from Sigma (St. Louis, MO). The antibodies used in this study were: anti-integrin α_v_(RMV-7) mAb [10], anti-integrin β_3 _(HMβ) mAb (BioLegend, San Diego CA) [11], anti-rabbit phospho ERK/ERK, anti-rabbit phospho JNK/JNK, and anti-rabbit phospho-p38 MAP kinase/p38 MAP kinase (New England Biolabs, Beverly, MA), and anti-phospho-focal adhesion kinase (FAK) (pY397)/anti-FAK (Santa Cruz Biotechnology, Inc., Santa Cruz, CA).

### Animal protocols

Specific, pathogen-free male BALB/C mice (Daehan Biolink Co. Eumsung-Gun, Chungbuk, Republic of Korea) weighing 19–21 g were used in all experiments. The Animal Care Committee of the Ewha Medical Research Institute approved the experimental protocol. Mice were cared for and handled in accordance with the National Institutes of Health (NIH) Guide for the Care and Use of Laboratory Animals.

Mouse pharyngeal aspiration was performed as described by Rao et al. [12]. Animals were anesthetized with a mixture of ketamine and xylazine (45 mg/kg and 8 mg/kg, *i.p.*, respectively). Test solution (30 μl) containing LPS (1.5 mg/kg) was placed posterior in the throat and aspirated into the lungs. Control mice were administrated sterile saline (0.9% NaCl). Animals were administered with RGDS or RGES peptide (1, 2.5 or 5 mg/kg, i.p.) once one hour before LPS treatment and sacrificed 4 h post-LPS [13,14]. Animals were also administered RGDS or RGES peptide (5 mg/kg, i.p.) once at different time points (1 h before or 2 h after LPS treatment) and sacrificed 24 h post-LPS. In addition, animals were administered with α_v_β_3_-blocking mAbs, anti-α_v_, or anti-β_3 _(5 mg/kg, i.p.) once 1 h before and sacrificed 4 h post-LPS. Animals administered with these mAbs 2 h after LPS treatment were sacrificed 24 h post-LPS [15].

### Isolation of bronchoalveolar lavage (BAL) cells, lung tissue, and cell counts

BAL was performed through a tracheal cannula using 0.8 ml aliquots of ice-cold Ca^2+^/Mg^2+^-free phosphate-buffered medium (145 mM NaCl, 5 mM KCl, 1.9 mM NaH_2_PO_4_, 9.35 mM Na_2_HPO_4_, and 5.5 mM dextrose; at pH 7.4) for a total of 2.4 ml for each mouse. Cell counts were determined using an electronic Coulter Counter fitted with a cell sizing analyzer (Coulter Model ZBI with a channelizer 256; Coulter Electronics, Bedfordshire, UK), as described by Lane and Mehta [16]. Neutrophils and macrophages were identified by their characteristic cell diameter [17].

### Measurement of total protein in BAL samples

BAL protein concentration was used as an indicator of blood-pulmonary epithelial cell barrier integrity [18]. Total protein was measured using the method devised by Hartree [19], using bovine serum albumin as a standard.

### TNF-α and macrophage inflammatory protein (MIP)-2 measurements

First BAL fluid was assayed using TNF-α and MIP-2 enzyme-linked immunosorbent assay (ELISA) kits (R&D Systems, Minneapolis, MN) according to the manufacturer's instructions. Concentrations of TNF-α, and MIP-2 were determined as picograms per milliliter based on the appropriate standard curve.

### Zymographic analysis of matrix metalloproteinase-9 (MMP-9)

The gelatinolytic activities of BAL fluid samples were determined by zymography using gelatin copolymerized with acrylamide, as described previously [20]. Clear zymogram bands were photographed using negative Polaroid 665 film. MMP-9 activity was quantified by densitometry using an UltroScan XL laser densitometer (LKB, Model 2222-020).

### Western blot analysis

Lung tissue homogenate was separated on 10% SDS-polyacrylamide gels. Separated proteins were electrophoretically transferred onto nitrocellulose paper and blocked for 1 h at room temperature with Tris-buffered SAL containing 3% BSA. Membranes were then incubated with the indicated Abs and visualized by chemiluminescence (ECL).

### Fibrinogen binding assay

Adherent cells (> 95% alveolar macrophages) were incubated in 100 μl DMEM containing 80 μg/ml fibrinogen conjugated to Alexafluor-488, 2 mM CaCl_2_, and 4 mg/ml BSA for 20 min [13]. Cells were then resuspended in a fixation solution. Nuclei were stained with Hoechst no. 33258 (5 μg/ml, Calbiochem) for 10 min and slides were then mounted. Images were collected using an LSM Image Examiner on a confocal laser scanning microscope (LSM-5 Pascal Exciter, Carl Zeiss, Germany).

### Lung histology

Lungs were fixed with 10% buffered formalin at room temperature for 48 h, dehydrated, and embedded in paraffin. Sections (4 μm) were stained with hematoxylin and eosin (H&E). Light microscopic analysis of lungs was performed by blinded observation.

### Statistical analysis

Values are expressed as means ± SEM. Inter-group comparisons were performed using one-way ANOVA followed by Tukey's *post hoc test*. Statistical significance was set at P < 0.05.

## Results

### Effects of RGDS peptide on the recruitment of inflammatory cells to lungs

**Figure 1:**
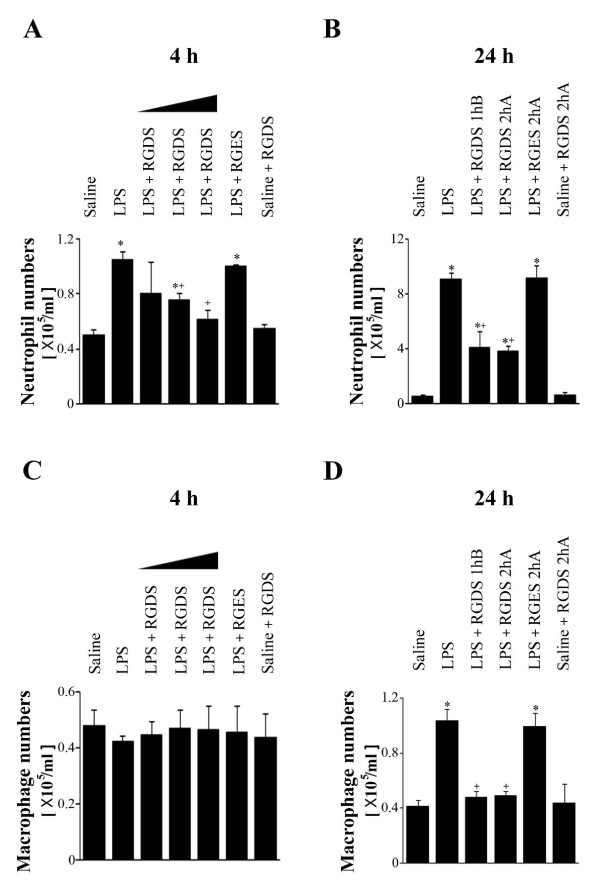
**Effects of RGDS peptide on LPS-induced increases in neutrophil (A, B) and macrophage (C, D) numbers in bronchoalveolar lavage (BAL) fluid**. Where indicated, mice were administered *i.p. *RGDS (1, 2.5 or 5 mg/kg) or RGES (5 mg/kg, a negative control) once 1 h before LPS treatment and sacrificed 4 h post-LPS. Mice were also administered these peptides (5 mg/kg, *i.p.*) once 1 h before or 2 h after LPS and sacrificed 24 h post-LPS. Values represent means ± SEM of 5 mice per group. *Significantly different from saline treated controls, p < 0.05; ^+^significantly different from animals treated with LPS only, p < 0.05.

The neutrophil cell numbers in BAL fluid increased at 4 and 24 h after *i.t. *LPS (1.05 ± 0.06 × 10^5^/ml and 9.10 ± 0.43 × 10^5^/ml, respectively) (Figures 1A and 1B). RGDS (1, 2.5, or 5 mg/kg, *i.p*., 1 h before LPS) inhibited these neutrophil increases 4 h post-LPS in a dose-dependent manner (Figure 1A). Peak inhibition of 79% was observed at 5 mg/kg. At 24 h post-LPS, we evaluated and compared the inhibitory effects of RGDS (5 mg/kg) on inflammatory cell migration when administered at different times, i.e., at 1 h before or 2 h after LPS (Figure 1B). Interestingly, the inhibitory effect of RGDS on LPS-induced neutrophil increases 24 h post-LPS was similar when administered at these times (58 and 62% vs. LPS treated animals, respectively).

Macrophage numbers in BAL fluid were unchanged at 4 h post-LPS (Figure 1C), but were increased 2.5-fold at 24 h post-LPS (1.04 ± 0.08 × 10^5^/ml) (Figure 1D), and RGDS treatment 1 h before or 2 h after LPS significantly inhibited this increase. Moreover, the inhibitory effects of RGDS were not significantly different (89 and 88% when administered at these times vs. LPS treated animals, respectively, *p *< 0.05) (Figure 1D). LPS+RGES (a negative control) or saline+RGDS did not affect neutrophil and macrophage cell numbers compared with LPS- or saline-treated mice, respectively.

**Figure 2:**
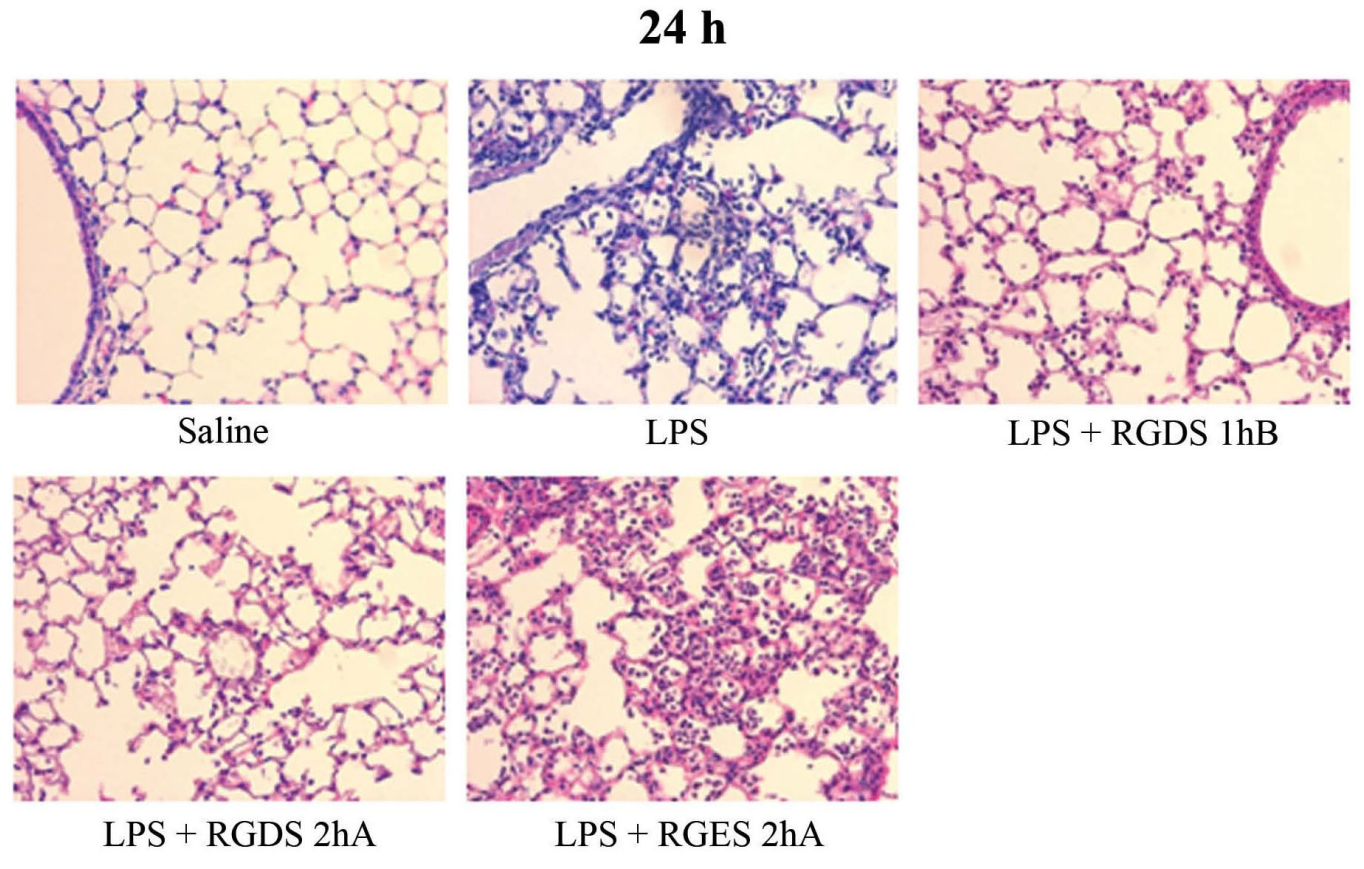
**Reduction in LPS-induced lung inflammation by RGDS**. Mice were administered RGDS (5 mg/kg, *i.p.*) once 1 h before or 2 h after LPS and were sacrificed 24 h post-LPS. Lung sections were H&E stained, as described in Methods. Representative staining from 5 mice per group are shown.

Histological lung sections obtained 24 h post-LPS confirmed BAL findings. H&E sections of lungs fixed with paraformaldehyde revealed similar reductions in peribroncheal and intraalveolar infiltration of neutrophils in lungs treated with RGDS at different times (Figure 2).

### Effect of RGDS on protein levels in BAL fluid

**Figure 3:**
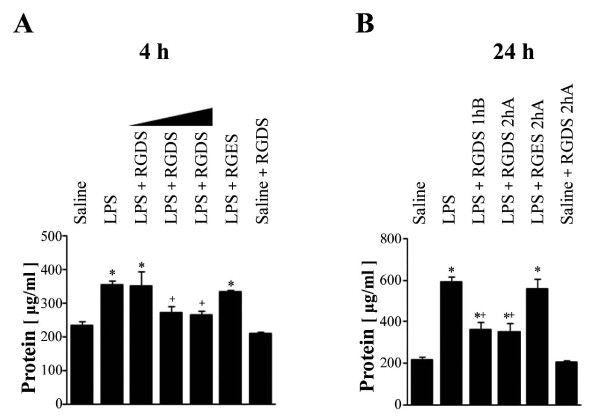
**Effects of RGDS on LPS-induced increases in total protein levels in BAL fluid**. Where indicated, mice were administered *i.p. *RGDS (1, 2.5 or 5 mg/kg) or RGES (5 mg/kg) once 1 h before LPS and sacrificed 4 h post-LPS (A). Mice were also administered these peptides (5 mg/kg, *i.p.*) once 1 h before or 2 h after LPS and were sacrificed 24 h post-LPS (B). Values represent means ± SEM of 5 mice per group. *Significantly different from saline treated controls, p < 0.05; ^+^significantly different from animals treated with LPS only, p < 0.05.

LPS treatment significantly increased protein content in BAL fluid at 4 and 24 h post-LPS, by 1.5- and 2.6-fold, respectively, compared with saline treated controls (Figures 3A and 3B). RGDS (2.5 or 5 mg/kg, 1 h before LPS) significantly decreased protein levels at 4 h post-LPS by 70 and 73%, respectively (*p *< 0.05) (Figure 3A). At 24 h post-LPS, RGDS treatment 1 h before or 2 h after LPS, similarly and significantly inhibited this LPS-induced BAL protein increase by 62, and 64% vs. LPS, respectively (*p *< 0.05) (Figure 3B). LPS+RGES or saline+RGDS had no effect on protein accumulation compared with LPS- or saline-treated mice, respectively.

### Effects of RGDS on TNF-α and MIP-2 levels, and MMP-9 activity

**Figure 4:**
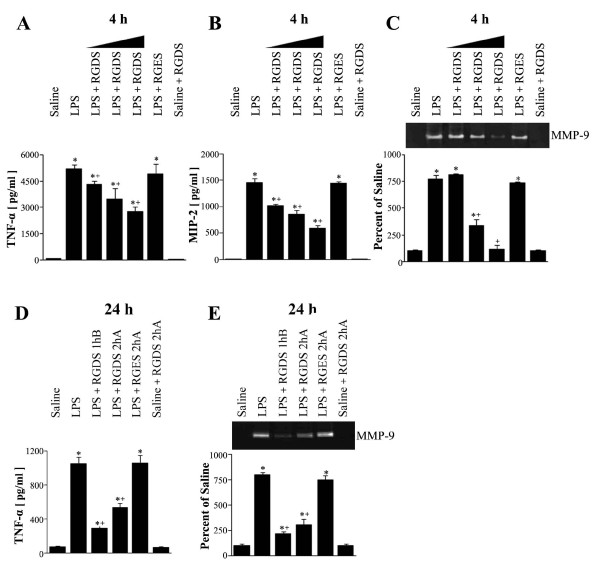
**Effects of RGDS on LPS-induced TNF-α and MIP-2 productions, and on MMP-9 activity**. Where indicated, mice were administered *i.p. *RGDS (1, 2.5 or 5 mg/kg) or RGES (5 mg/kg) once 1 h before LPS treatment and sacrificed 4 h post-LPS. Mice were also administered these peptides (5 mg/kg, *i.p.*) once 1 h before or 2 h after LPS and were sacrificed 24 h post-LPS (B). TNF-α and MIP-2 levels in BAL fluid samples were quantified using enzyme-linked immunosorbent assays (A, B, D). Gelatinolytic activity in BAL fluid samples (C, E). Samples were analyzed by zymography followed by scanning densitometry. The 92-kD genolytic bands corresponded to MMP-9. Densities are expressed as percentages versus saline treated controls. Values represent means ± SEM of results from 5 mice per group. *Significantly different from saline treated controls, p < 0.05; ^+^significantly different from animals treated with LPS only, p < 0.05.

TNF-α, MIP-2, and MMP-9 are representative proinflammatory mediators, which play major roles in neutrophil influx and lung damage. TNF-α production and MMP-9 expression require integrin signaling, as demonstrated by in vitro inflammatory cell models [21-26]. Here, we evaluated the effects of RGDS on these proinflammatory mediators in BAL fluid. As shown in Figures 4A and 4B, RGDS administered 1 h before LPS inhibited LPS-induced increases in TNF-α and MIP-2 levels in BAL fluid at 4 h post-LPS. Moreover, these inhibitions were in dose-dependent and peaked at 5 mg/kg when these inhibitions of TNF-α and MIP-2 levels were 47 and 60%, respectively. At 24 h post-LPS, LPS-induced TNF-α levels in BAL fluid were decreased by a fifth of those at 4 h post-LPS and MIP-2 levels were decreased to saline control (data not shown). Pre- or posttreatment with RGDS (5 mg/kg) significantly inhibited LPS-induced TNF-α levels by 78 and 53%, respectively (Figure 4D).

BAL fluid was also analyzed for evidence of MMP-9 activity by gelatin zymography. MMP-9 activity increased 7.7- and 8.0-fold vs saline treated controls at 4 and 24 h post-LPS, respectively. RGDS (2.5 or 5 mg/kg, 1 h before LPS) significantly inhibited LPS-induced MMP-9 activity in BAL fluid 4 h post-LPS (by 59 and 91%, respectively (*p *< 0.05) (Figure 4C). Pre- or posttreatment with RGDS (5 mg/kg) significantly inhibited LPS-induced MMP-9 activity by 83 and 70%, respectively, 24 h post-LPS (Figure 4E).

TNF-α and MIP-2 levels and MMP-9 activity were similar in samples obtained from LPS and LPS-RGES treated animals at 4 or 24 h post-LPS.

### Effects of RGDS on the activation of integrin signaling and MAP kinases

**Figure 5:**
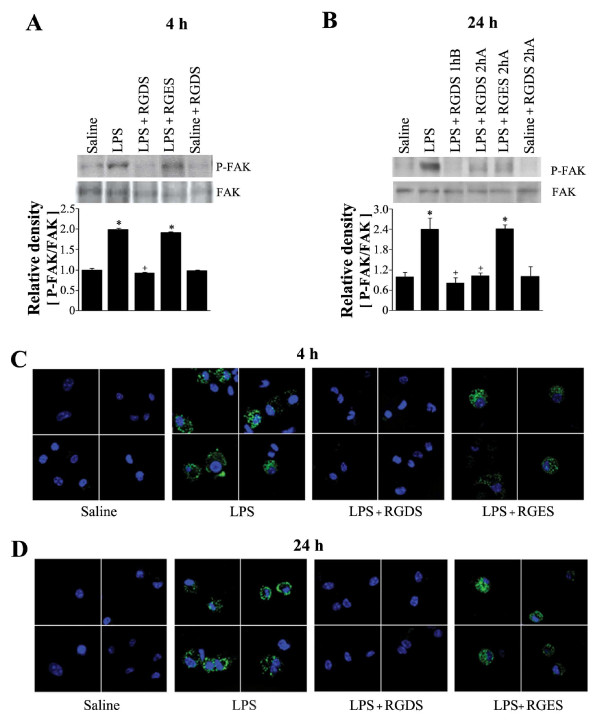
**Effects of RGDS on FAK phosphorylation in lung tissue (A, B), and fibrinogen binding activity in alveolar macrophages (C, D)**. Where indicated, mice were administered RGDS or RGES (5 mg/kg, *i.p.*) once 1 h before LPS treatment and sacrificed 4 h post-LPS. Mice were also administered this peptide (5 mg/kg, *i.p.*) once 1 h before or 2 h after LPS and sacrificed 24 h post-LPS. (A, B) Western blotting with each anti-specific (phospho) FAK Ab was performed on lung tissue homogenates. Relative values for phosphorylated FAK versus FAK are indicated below the gel. Values represent means ± SEM of 5 mice per group. *Significantly different from saline treated controls, p < 0.05; ^+^significantly different from animals treated with LPS only, p < 0.05. (C, D) Alveolar macrophages were incubated with fibrinogen conjugated to Alexafluor-488 for 20 min. After exposure to fibrinogen for 20 min, cells were fixed and then analyzed by confocal fluorescence microscopy. The results are representative results obtained from 5 mice per group.

In vitro inhibition of integrin signaling by RGD peptide has been reported to decrease LPS-induced MAP kinase activity and TNF-α production in macrophages [9]. To investigate the functional link between integrin signaling and LPS-induced MAP kinase activation during the development of acute lung injury, we evaluated the inhibitory effect of RGDS on LPS-induced FAK phosphorylation in lung tissue. RGDS treatment 1 h before LPS completely inhibited LPS-induced this kinase phosphorylation at 4 h post-LPS (Figure 5A). At 24 h post-LPS, pre- or posttreatment with RGDS also completely inhibited LPS-induced FAK phosphorylation with similar potencies (Figure 5B). We also examined the fibrinogen binding activity of alveolar macrophages, because fibrinogen is a specific and physiologic ligand for activated integrin complexes, including α_v_β_3 _[27]. As was expected, alveolar macrophages from mice treated with LPS were found to have increased fibrinogen binding activity in many confocal microscope fields, and RGDS treatment 1 h before or 2 h after LPS suppressed this activity at 4 or 24 h post-LPS, respectively (Figures 5C and 5D). These findings indicate that RGDS inhibits LPS-induced integrin signaling during the development of lung injury.

**Figure 6:**
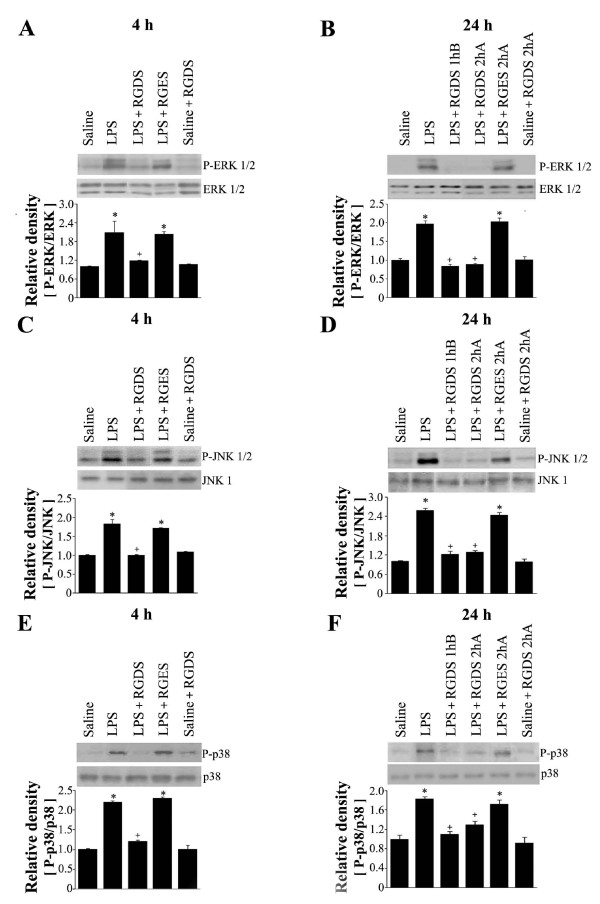
**Effects of RGDS on phosphorylation of ERK1/2 (A, B), JNK1/2(C, D) and p38 MAP kinase (E, F) in lung tissue**. Where indicated, mice were administered RGDS or RGES (5 mg/kg, *i.p.*) once 1 h before LPS treatment and sacrificed 4 h post-LPS. Mice were also administered these peptides (5 mg/kg, *i.p.*) once 1 h before or 2 h after LPS and sacrificed 24 h post-LPS. Western blotting was performed with each anti-specific (phospho) Ab on lung tissue homogenates. Relative values for phosphorylated MAP kinase versus MAP kinase, respectively, are indicated below the gel. Values represent means ± SEM of 5 mice per group. *Significantly different from saline treated controls, p < 0.05; ^+^significantly different from animals treated with LPS only, p < 0.05.

Figure 6 presents LPS-induced MAP kinase phosphorylation data in lung tissue at 4 or 24 h post-LPS, which concurs with the findings of our previous study [28,29]. Phosphorylation of ERK and JNK substantially were increased 4 h post-LPS, and were either maintained (ERK) (Figures 6A and 6B) or further increased (JNK) (Figures 6C and 6D). p38 MAP kinase phosphorylation substantially increased at 4 h and this phosphorylation level was slightly reduced 24 h post-LPS (Figures 6E and 6F). RGDS treatment 1 h before LPS significantly suppressed LPS-induced phosphorylation of ERK, JNK and p38 MAP kinase 4 h post-LPS. Pre- or posttreatment with RGDS also inhibited all three MAP kinase phosphorylation 24 h post-LPS. The smallest decrease was observed for p38 MAP kinase. These findings suggest that RGD motif-dependent integrin signaling, involving FAK activation, is solidly linked to LPS-induced ERK and JNK activations during the progression of acute lung injury, and that it is weakly linked to p38 MAP kinase activation.

### Involvement of integrin α_v_β_3 _in the inflammatory responses

**Figure 7:**
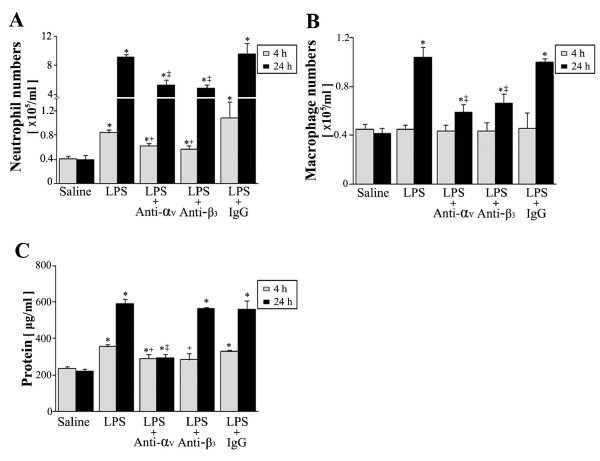
**Effects of anti-α_v _or anti-β_3 _mAb on LPS-induced increases in inflammatory cell numbers and total protein levels in BAL fluid**. Where indicated, mice were administered anti-α_v_, anti-β_3_, or IgG (5 mg/kg, *i.p.*) once 1 h before LPS treatment and sacrificed 4 h post-LPS. Mice were also administered these mAbs (5 mg/kg, *i.p.*) once 2 h after LPS and sacrificed 24 h post-LPS. Neutrophil (A) and macrophage (B) numbers and total protein levels (C) in BAL fluid. Values represent means ± SEM of 5 mice per group. *Significantly different from saline treated controls, p < 0.05; significantly different at 4 h (^+^) and 24 h (^‡^) post-LPS from animals treated with LPS only, p < 0.05.

The findings of the present study suggest the involvements of RGD motif-dependent integrin binding and integrin signal activation during the initiation and progression of LPS-induced lung injury in our mouse model. The RGD motif binds specifically to α_v_β_3 _integrins expressed on the surface of macrophages, neutrophils, endothelial cells, and migrating smooth muscle cells [30,31]. However, no data is available on the roles of integrin α_v_β_3 _in leukocyte migration to the lungs or in other pulmonary inflammatory responses. Therefore, we evaluated the involvement of RGD motif-dependent integrin α_v_β_3 _binding in the key pulmonary inflammatory responses to LPS treatment using a blocking anti-α_v _or anti-β_3 _mAb. Anti-α_v_or anti-β_3 _(5 mg/kg, *i.p*., 1 h before LPS) significantly inhibited the neutrophil number increases in BAL fluid by 50 and 61% at 4 h post-LPS (*p *< 0.05), respectively (Figure 7A). Similarly, at 24 h post-LPS, anti-α_v _or anti-β_3 _at 2 h after LPS significantly inhibited neutrophil increases (by 44 and 50%, respectively, *p *< 0.05) (Figure 7A) and macrophage increases (by 71 and 60%, respectively, *p *< 0.05) (Figure 7B).

Moreover, anti-α_v _(1 h before or 2 h after LPS) significantly inhibited LPS-induced BAL protein increases at 4 and 24 h post-LPS by 53 and 80%, respectively (Figure 7C). Interestingly, the inhibitory effect of anti-β_3 _mAb was effective only at 4 h post-LPS (59% inhibition), but it was not effective at 24 h post-LPS (7% inhibition).

**Figure 8:**
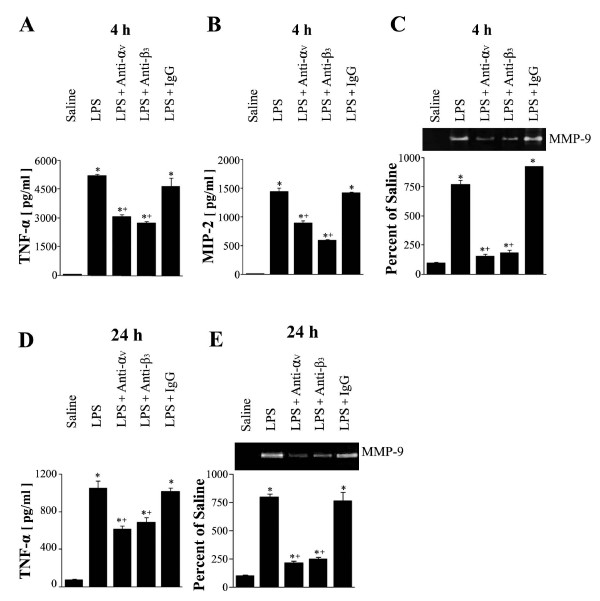
**Effects of anti-α_v _or anti-β_3 _mAb on LPS-induced TNF-α and MIP-2 productions, and on MMP-9 activity**. Where indicated, mice were administered anti-α_v_, anti-β_3_, or IgG (5 mg/kg, *i.p.*) once 1 h before LPS treatment and sacrificed 4 h post-LPS. Mice were also administered these mAbs (5 mg/kg, *i.p.*) once 2 h after LPS and sacrificed 24 h post-LPS. TNF-α(A, D) andMIP-2 (B) productions, and MMP-9 activity (C, E) in BAL fluid. Values represent means ± SEM of 5 mice per group. *Significantly different from saline treated controls, p < 0.05;^+^significantly different from animals treated with LPS only, p < 0.05.

LPS-induced increases in TNF-α and MIP-2 levels in BAL fluid 4 h post-LPS were reduced by anti-α_v _(by 42 and 39%, respectively) or anti-β_3 _mAb (by 48 and 60%, respectively) when administered 1 h before LPS (*p *< 0.05) (Figure 8A, 8B). At 24 h post-LPS, TNF-α levels were also inhibited by posttreatment with anti-α_v _or anti-β_3 _(45 and 38% inhibition, respectively) (Figure 8D). Anti-α_v _or anti-β_3 _inhibited LPS-induced MMP-9 activity in BAL fluid at 4 h post-LPS by 87 and 82% and at 24 post-LPS by 73 and 79%, respectively (Figure 8C and 8E).

## Discussion

In the present study, we determined: (1) RGDS effectively inhibited LPS-induced inflammatory cell migration into the lung, protein accumulation and proinflammatory mediator production in BAL fluid; (2) RGDS inhibited LPS-induced activations of integrin signaling and MAP kinases in lung tissue; (3) anti-α_v _mAb inhibited LPS-induced inflammatory cell migration into the lung, protein accumulation and proinflammatory mediator production in BAL fluid; (4) anti-β_3 _was also effective at inhibiting LPS-induced inflammatory responses, except protein leakage.

Small-molecule RGD peptides have been known to provide specificity as well as high affinity for RGD recognizing integrins [32]. Previously, RGD-containing synthetic peptides have been shown to inhibit leukocyte recruitment and inflammatory mediator production in arthritis [33] and liver injury [8]. However, the effect of synthetic RGDS peptide on the pulmonary inflammatory responses is not evaluated. In the present study, we demonstrate that synthetic RGDS peptide can be used to inhibit pulmonary inflammatory responses to *i.t. *LPS. Pre- or posttreatment with RGDS significantly inhibited neutrophil and alveolar macrophage numbers in BAL fluid at 4 or 24 h post-LPS. In addition, the lung histologic findings clearly demonstrated that RGDS effectively reduced LPS-induced lung parenchymal infiltration of neutrophils with similar potency when it was administered before or after LPS. Like RGDS, anti-α_v _or anti-β_3 _mAb blocking of integrin α_v_β_3 _ligation markedly inhibited LPS-induced increases in neutrophil and alveolar macrophage numbers in BAL fluid at 4 or 24 h post-LPS. To the best of our knowledge, this provides first evidence that integrin α_v_β_3_is utilized for migration to regions of lung inflammation by both cell types. Since α_v_β_3 _is one of major integrin receptors binding to the RGD motifs of extracellular matrix (ECM) proteins, these findings suggest that RGDS specifically inhibits integrin (α_v_β_3_)-dependent inflammatory cell recruitment to inflamed pulmonary sites. Moreover, this inhibitory effect of RGDS is β_2_integrin-independent, because it does not block β_2 _integrins [23].

Another important finding made in the present study was that pre- or posttreatment with RGDS significantly inhibited LPS-induced increases in total protein in BAL fluid at 4 and 24 h post-LPS. Similarly, pre- or posttreatment with anti-α_v _significantly inhibited total protein level increases in BAL fluid as potent as RGDS at both time points, while anti-β_3 _was not effective at 24 h post-LPS. Although BAL protein is not a very specific way to measure pulmonary permeability, this discrepancy suggests that prevention of pulmonary edema and other inflammatory responses, such as leukocyte migration and inflammatory mediator production, are regulated by different mechanisms [34], and that blockade of the α_v _integrins (e.g. α_v_β_5 _and/or α_v_β_6_) other than α_v_β_5 _is likely to be responsible for the protective effects of RGDS on BAL protein accumulation. Indeed, a blocking antibody against the integrin α_v_β_5 _or α_v_β_6 _protected development of lung vascular permeability in different models of acute lung injury [35-37]. Additional studies are required to identify a physiological role of β_3 _integrin in the development of pulmonary edema during acute lung injury.

The productions of TNF-α and chemokines are essentially required for early LPS-mediated neutrophil recruitment and lung damage. Moreover, MMPs, like MMP-9, are required to allow activated neutrophils to transverse ECM barriers after adhesion, and to enable transendothelial cell migration. Therefore, RGDS may protect against LPS-induced acute lung injury by decreasing the productions or activities of these proinflammatory mediators. According to this concept, our data indicate RGDS significantly inhibited LPS-induced TNF-α and MIP-2 productions and MMP-9 activity in BAL fluid at 4 or 24 h post-LPS. Although its inhibitory effects on TNF-α production and MMP-9 activity were more potent in the pretreatment model compared with the posttreatment model at 24 h post-LPS, the inhibition was evident when it was administered 2 h after LPS. Similarly, pre- or posttreatment with anti-α_v _or anti-β_3 _also blocked LPS-induced TNF-α or MIP-2 levels and MMP-9 activity in BAL fluid at 4 or 24 h post-LPS as potently as RGDS. Our results suggest that the inhibitory effect of RGDS on LPS-induced these inflammatory mediator productions or activity during the development of acute lung injury is α_v_β_3 _integrin-dependent.

These proinflammatory mediators are known to be regulated at the transcriptional level by MAP kinase pathways and nuclear factor kappa B (NF-κB) [38-41]. Previously, we demonstrated that the RGD motifs of ECM entities that associate with integrin α_v_β_3 _signaling appears to be involved in NF-κB activation during acute lung injury [13]. In the present study, we report that integrin signaling-dependent MAP kinase activations are associated with LPS-induced acute lung injury. The regulation of multiple cellular events in the context of in vivo LPS stimulation is complex. Because of the extreme bioactivity of many molecules (i.e., TNF-α) produced by inflammatory cells [9], these may affect optimal LPS signaling to MAP kinase activation correlating with kinetics at many levels. Previously, we demonstrated the kinetics of LPS-induced phosphorylation of these kinases in lung tissue. MAP kinase phosphorylation beginning 2 h after LPS, substantially increased at 4 h and progressively further increased (JNK activation), were maintained (ERK activation), or decreased (p38 MAP kinase activation) for up to 24 h after LPS [28,29], which concurs with the kinetic results of our present study. Pre- or posttreatment with RGDS markedly inhibited LPS-induced phosphorylation of these MAP kinases in lung tissues at 4 or 24 h post-LPS, although it was less effective at inhibiting p38 MAP kinase phosphorylation 24 h post-LPS. In-line with our in vivo data, LPS signaling activated the integrin signal that amplifies ERK and JNK activations in macrophages [42]. Moreover, inhibition of LPS-induced cell adhesion and integrin signaling by RGD resulted in reductions in these MAP kinase activities in the order ERK ≈ JNK > p38 MAP kinase [9]. Previously, we found that inhibition of each of these MAP kinases reduced the LPS-induced proinflammatory mediator productions during acute lung injury [28,29]. Furthermore, inhibition of these kinases, except ERK, reduced LPS-induced phosphorylation and degradation of IκB-α and NF-κB activation. Collectively, these findings suggest that the specific inhibition of integrin signaling by RGDS results in suppression of proinflammatory mediator productions through the MAP-kinase leading to NF-κB pathway or each different pathway.

Integrin-mediated adhesion and/or clustering led to enhance the tyrosine phosphorylation of Src tyrosine kinases and FAK [43-45], and this pathway activates the classic Ras-MEK-ERK cascade [46]. We do suggest the involvement of a pathway initiated by FAK activation through integrin (α_v_β_3_) signaling during LPS-induced acute lung injury, because RGDS suppressed LPS-induced FAK phosphorylation in lung tissue and fibrinogen binding activity in alveolar macrophages. Indeed, anti-α_v _or anti-β_3 _mAb inhibited LPS-induced c-Src and FAK activation during acute lung injury [13]. Integrin-FAK-ERK/-JNK pathways have been documented in other in vitro systems [47-49].

## Conclusion

The present study suggests that RGDS inhibits integrin (α_v_β_3_)-dependent induction of inflammatory cell migration into the lungs, and proinflammatory mediator production. Our data also suggest that the protective effect of RGDS on pulmonary leakage is α_v_-dependent, but not β_3_. Importantly, posttreatment with RGDS was also highly effective at reducing these inflammatory responses correlated with its inhibitory effect on integrin signaled MAP kinase pathways. In addition, blockading the integrin-FAK-MAP kinase pathway with RGDS may reduce lung injury progression. Since RGDS has high specificity for α_v_integrins and the *in vivo *efficacy to access target cells as a small-molecule [8,32,50], it may effectively attenuate inflammatory cascade during LPS-induced development of acute lung injury.

## Abbreviations

RGDS: Arg-Gly-Asp-Ser; RGES: Arg-Gly-Glu-Ser; LPS: lipopolysaccharide; MAP: mitogen-activated protein; BAL: bronchoalveolar lavage; TNF-α: tumor necrosis factor-α; FAK: focal adhesion kinase; MIP: macrophage inflammatory protein; MMP-9: matrix metalloproteinase; ECM: extracellular matrix; NF-κB: nuclear factor kappa B.

## Competing interests

The authors declare that they have no competing interests.

## Authors' contributions

CM, JRH, HJP, and JH carried animal studies. CM performed the statistical analysis. JLK conceived of the study, participated in its design and coordination, and drafted and edited the manuscript. All authors read and approved the final manuscript.

## References

[B1] Ware LB, Mattay MA (2000). The acute respiratory distress syndrome. N Engl J Med.

[B2] Buckley CD, Doyonnas R, Newton JP, Blystone SD, Brown EJ, Watt SM, Simmons DL (1996). Identification of α_v_β_3 _as a heterotypic ligand for CD31/PECAM-1. J Cell Sci.

[B3] Kao WJ, Lee D (2001). In vivo modulation of host response and macrophage behavior by polymer networks grafted with fibronectin-derived biomimetic oligopeptides: the role of RGD and PHSRN domains. Biomaterials.

[B4] Sage EH (2001). Regulation of interactions between cells and extracellular matrix command performance on several stages. Clin Invest.

[B5] Chung A, Gao Q, Kao WJ (2007). Macrophage matrix metalloproteinase-2/-9 gene and protein expression following adhesion to ECM-derived multifunctional matrices via integrin complexation. Biomaterials.

[B6] Bengtsson T, Grenegård M (2002). Leucocyte activation by collagen-stimulated platelets in whole blood. Scand J Clin Lab Invest.

[B7] Koning GA, Schiffelers MR, Wauben MHM, Kok RJ, Mastrobattista E, Molema G, ten Hagen TLM, Storm G (2006). Targeting of angiogenic endothelial cells at sites of inflammation by dexamethasone phosphate-containing RGD peptide liposomes inhibits experimental arthritis. Arthritis Rheum.

[B8] Fondevila C, Shen XD, Moore C, Busuttil RW, Coito AJ (2005). Cyclic RGD peptides with high affinity for α_5_β_1 _integrin protect genetically fat Zucker rat livers from cold ischemia/reperfusion injury. Transplantation Proceedings.

[B9] Monick MM, Powers L, Butler N, Yarovinsky T, Hunninghake GW (2002). Interaction of matrix with integrin receptors is required for optimal LPS-induced MAP kinase activation. Am J Physiol Lung Cell Mol Physiol.

[B10] Narumiya S, Abe Y, Kita Y, Miyake K, Nakajima K, Watanabe TX, Oka Y, Sugiyama H, Yagita H, Okumura K, Toshiyuki H, Fujiware H (1994). Pre-B cells adhere to fibronectin via interactions of integrin alpha 5/alpha V with RGDS as well as of integrin alpha 4 with two distinct V region sequences at its different binding sites. Int Immunol.

[B11] Piali L, Hammel P, Uherek C, Bachmann F, Gisler RH, Dunon D, Imhof BA (1995). CD31/PECAM-1 is a ligand for alpha v beta 3 integrin involved in adhesion of leukocytes to endothelium. J Cell Biol.

[B12] Rao GVS, Tinkle S, Weissman DN, Antonini JM, Kashon ML, Salmen R, Battelli LA, Willard PA, Hoover MD, Hubbs AF (2003). Efficacy of a technique for exposing the mouse lung to particles aspirated from the pharynx. J Toxicol Environ Health A.

[B13] Lee HS, Moon C, Lee HW, Park EM, Cho MS, Kang JL (2007). Src tyrosine kinases mediate activations of NF-κB and integrin signal during lipopolysaccharide-induced acute lung injury. J Immunol.

[B14] Obergfell A, Eto K, Mocsai A, Buensuceso C, Moores SL, Brugge JS, Lowell CA, Shattil SJ (2002). Coordinate interactions of Csk, Src, and Syk kinases with αIIβ3 initiate integrin signaling to the cytoskeleton. J Cell Biol.

[B15] Bendeck MP, Nakada MT (2001). The β3 integrin antagonist m7E3 reduces matrix metalloproteinase activity and smooth muscle cell migration. J Vasc Res.

[B16] Lane FC, Mehta JR (1990). In vitro human tumor sensitivity assay using cell counting and sizing. Am Biotechnol Lab.

[B17] Castranova V, Jones T, Afshari MWA, Frazer DJ, Jacobs RR, Wakelyn PJ, Domelsmith LN (1990). Pulmonary responses of guinea pigs to consecutive exposures to cotton dust. Proceedings of the 14th Cotton Dust Research Conference.

[B18] Beck BD, Brain JD, Bohannon DE (1982). An in vitro hamster bioassay to assess the toxicity of particulates for the lung. Toxicol Appl Pharmacol.

[B19] Hartree EF (1972). Determination of protein: a modification of the Lowry method that gives a linear photometric response. Anal Biochem.

[B20] Kang JL, Lee HW, Lee HS, Pack IS, Chong Y, Castranova V, Koh Y (2001). Genistein prevents nuclear factor-kappa B activation and acute lung injury induced by lipopolysaccharide. Am J Respir Crit Care Med.

[B21] Huveneers S, Truong H, Danene HJ (2007). Integrins: Signaling, disease, and therapy. Int J Radiat Biol.

[B22] Dellacasagrande J, Ghigo E, Hammami SM, Toman R, Raoult D, Capo C, Mege JL (2000). alpha(v)beta(3) integrin and bacterial lipopolysaccharide are involved in Coxiella burnetii-stimulated production of tumor necrosis factor by human monocytes. Infec Immun.

[B23] Kwak SH, Mitra S, Bdeir K, Strassheim D, Park JS, Kim JY, Idell S, Cines D, Abraham E (2005). The kringle domain of urokinase-type plasminogen activator potentiates LPS-induced neutrophil activation through interaction with {alpha}V{beta}3 integrins. J Leukocyte Biol.

[B24] Weber GF, Zawaideh S, Hikita S, Kumar VA, Cantor H, Ashkar S (2002). Phosphorylation-dependent interaction of osteopontin with its receptors regulates macrophage migration and activation. J Leukocyte Biol.

[B25] Kim CH, Lee KH, Lee CT, Kim YW, Han SK, Shim YS, Yoo CG (2004). Aggregation of β2 integrins activates human neutrophils through the IκB/NF-κB pathway. J Leukocyte Biol.

[B26] Zhu P, Xiong W, Rodgers G, Qwarnstrom EE (1998). Regulation of interleukin 1 signaling through integrin binding and actin reorganization: disparate effects on NF-κB and stress kinase pathways. Biochem J.

[B27] Liaw L, Lindner V, Schwartz SM, Chambers AF, Giachelli CM (1995). Osteopontin and beta 3 integrin are coordinately expressed in regenerating endothelium in vivo and stimulate Arg-Gly-Asp-dependent endothelial migration in vitro. Circ Res.

[B28] Lee HS, Kim HJ, Moon CS, Chong YH, Kang JL (2004). Inhibition of c-Jun NH_2_-terminal kinase or extracellular signal-regulated kinase improves lung injury. Respir Res.

[B29] Kim HJ, Lee HS, Chong YH, Kang JL (2006). p38 mitogen-activated protein kinase up-regulates LPS-induced NF-κB activation in the development of lung injury and RAW 264.7 macrophages. Toxicol.

[B30] Eliceiri BP, Cheresh DA (1998). The role of alphav integrins during angiogenesis. Mol Med.

[B31] Flier A van der, Sonnenberg A (2001). Function and interaction of integrins. Cell Tissue Res.

[B32] Takagi J (2004). Structural basis for ligand recognition by RGD (Arg-Gly-Asp)-dependent integrins. Biochem Society Transactions.

[B33] Wahl SM, Allen JB, Hines KL, Imamichi T, Wahl AM, Fucht LT, McCarthy JB (1994). Synthetic fibronectin peptides suppress arthritis in rats by interrupting leukocyte adhesion and recruitment. J Clin Invest.

[B34] Yamamoto T, Kajikawa O, Martin TR, Sharar SR, Harlan JM, Winn RK (1998). The role of leukocyte emigration and IL-8 on the development of lipopolysaccharide-induced lung injury in rabbits. J Immunol.

[B35] Jenkins RG, Su X, Su G, Scotton CJ, Camerer E, Laurent GJ, Davis GE, Chambers RC, Matthay MA, Sheppard D (2006). Ligation of protease-activated receptor a enhances α_v_β_6 _integrin-dependent TGF-β activation and promotes acute lung injury. J Clin Invest.

[B36] Pittet JF, Griffiths MJD, Geiser T, Kaminski N, Dalton SL, Huang X, Brown LAS, Gotwals PJ, Koteliansky VE, Matthay MA, Sheppard D (2001). TGF-β is a critical mediator of acute lung injury. J Clin Invest.

[B37] Su G, Hodnett M, Wu N, Atakilit A, Kosinski C, Godzich M, Huang XZ, Kim JK, Frank JA, Matthay MA, Sheppard D, Pittet JF (2007). Integrin αvβ5 regulates lung vascular permeability and pulmonary endothelial barrier function. Am J Respir Cell Mol Biol.

[B38] Bhattacharyya A, Pathak S, Datta S, Chattopadhyay S, Basu J, Kundu M (2002). Mitogen-activated protein kinases and nuclear factor-kappa B regulate Helicobacter pylori-mediated interleukin-8 release from macrophages. Biochem J.

[B39] Blackwell TS, Blackwell TR, Holden EP, Christman BW, Christman JW (1996). In vivo antioxidant treatment suppresses nuclear factor-kappa B activation and neutrophilic lung inflammation. J Immunol.

[B40] Kumar A, Dhawan S, Mukhopadhyay A, Aggarwal BB (1999). Human immunodeficiency virus-1-tat induces matrix metalloproteinase-9 in monocytes through protein tyrosine phosphatase-mediated activation of nuclear transcription factor NF-kappaB. FEBS Lett.

[B41] Scherle PA, Jones EA, Favata MF, Daulerio AJ, Covington MB, Nurnberg SA, Magolda RL, Trzaskos JM (1998). Inhibition of MAP kinase kinase prevents cytokine and prostaglandin E2 production in lipopolysaccharide-stimulated monocytes. J Immunol.

[B42] Perera PY, Mayadas TN, Takeuchi O, Akira S, Zaks-Zilberman M, Goyert SM, Vogel SN (2001). CD11b/CD18 acts in concert with CD14 and Toll-like reeptor (TLR) 4 to elicit full lipopolysaccharide and taxol-inducible gene expression. J Immunol.

[B43] Kaplan KB, Swedlow JR, Morgan DO, Varmus HE (1995). c-Src enhances the spreading of src-/- fibroblasts on fibronectin by a kinase-independent mechanism. Genes Dev.

[B44] Sawhney RS, Cookson MM, Omar Y, Hauser J, Brattain MG (2006). Integrin α2-mediated ERK and calpain activation play a critical role in cell adhesion and motility via focal adhesion kinase signaling. J Biol Chem.

[B45] Schaller MD, Borgman CA, Cobb BS, Vines RR, Reynolds AB, Parsons JT (1992). pp125FAK a structurally distinctive protein-tyrosine kinase associated with focal adhesions. Proc Natl Acad Sci USA.

[B46] Schlaepfer DD, Hauck CR, Sieg DJ (1999). Signaling through focal adhesion kinase. Prog Biophys Mol Biol.

[B47] Manohar A, Shome SG, Lamar J, Stirling L, Iyer V, Pumiglia K, DiPersio CM (2004). Alpha 3 beta 1 integrin promotes keratinocyte cell survival through activation of a MEK/ERK signaling pathway. J Cell Sci.

[B48] Sekimoto H, Eipper-Mains J, Pond-Tor S, Boney CM (2005). αvβ3 integrins and Pyk2 mediate insulin-like growth factor I activation of Src and nitrogen-activated protein kinase in 3T3-L1 cells. Mol Endocrinology.

[B49] Takino T, Nakada M, Miyamori H, Watanabe Y, Sato T, Gantulga D, Yoshioka K, Yamada KM, Sato H (2005). JSAP1/JIP3 cooperates with focal adhesion kinase to regulate c-Jun N-terminal kinase and cell migration. J Biol Chem.

[B50] Koivunen E, Wang B, Ruoslahti E (1995). Phage'libraries displacing cyclic peptides with different ring sizes: ligand specificities of the RGD-directed integrins. Biotechnology (NY).

